# Effects of poly-γ-glutamic acid and poly-γ-glutamic acid super absorbent polymer on the sandy loam soil hydro-physical properties

**DOI:** 10.1371/journal.pone.0245365

**Published:** 2021-01-12

**Authors:** Jianzhong Guo, Wenjuan Shi, Jiake Li, Zhongmin Zhai

**Affiliations:** State Key Laboratory of Eco-hydraulics in Northwest Arid Region of China, Xi’an University of Technology, Xi’an, China; Institute of Soil Science, CHINA

## Abstract

The main forms of poly-γ-glutamic acid (γ-PGA) applied in agriculture include agricultural γ-PGA and γ-PGA super absorbent polymer (SAP). Laboratory experiments were conducted with a check treatment CK (no γ-PGA added) and two different forms of γ-PGA added to sandy loam soil (T and TM stand for γ-PGA and γ-PGA SAP) at four different soil mass ratios (0.05% (1), 0.10% (2), 0.15% (3) and 0.20% (4)) to determine their effects on sandy loam soil hydro-physical properties. Both of them could reduce the cumulative infiltration of soil water. The total available water (TAW) which the soil water content (SWC) from field water capacity (FC) to permanent wilting point (PWP) after γ-PGA added into sandy loam soil had no significant different compared with CK, and the TAW was highest at the treatment of γ-PGA with 0.10% addition amount into sandy loam soil. However, the TAW of sandy loam soil increased dramatically with the γ-PGA SAP addition amount increasing. TM3 had the highest soil water absorption among the treatments with γ-PGA SAP. The T1 to T4 treatments with γ-PGA addition slightly prolonged retention time (RT) when SWC varied from FC to PWP compared with CK. For γ-PGA SAP addition treatments, the time for SWC varied from FC to PWP was 1.48 times (TM1), 1.88 times (TM2), 2.01 times (TM3) and 2.87 times (TM4) longer than that of CK, respectively. The results of this study will provide further information for the use of these materials in agricultural application.

## Introduction

Poly-γ-glutamic acid (γ-PGA) accounts for the polypeptide formed by glutamic acid monomer linked with the amide bonds across α-amino groups together with γ-carboxylic [1]. It is an environmentally friendly material due to its biodegradability, water-solubility, and naturality polymer [2]. γ-PGA and its derivatives have been paid more attention due to its easily modified structure [3]. It has application in the fields of medicine [4], chemical industry and environmental protection [5] due to it being a non-petroleum resource. γ-PGA has been fermented by the soil microorganism *Bacillus subtilis* using pig (*Sus scrofa domesticus*) manure, cow (*Bos taurus*) manure, and soybean (*Glycine max* L.) cake powders to be the major sources of nitrogen and carbon, all of which are the biological waste and renewable resources [6]. Hence, the cost of γ-PGA production can be greatly reduced. The decreasing price of γ-PGA makes the application of γ-PGA SAP in agricultural field possible. At present, γ-PGA and γ-PGA SAP have not been widely used in agricultural field.

γ-PGA produced by microbial fermentation can be used as a synergistic fertilizer in agriculture after filtering and crude purification [7]. γ-PGA applied to wheat (*Triticum aestivum* L.) grown in pots increased yield, nitrogen use efficiency, and soil microorganisms [8]. γ-PGA applied to maize (*Zea mays* L.) that was planted within pots could increase survival rate at drought conditions and biomass, and promote growth of roots and leaves [9]. The application of γ-PGA in a cotton (*Gossypium hirsutum* L.) field experiments in Xinjiang, China could increase cotton yield and improve cotton fiber quality [10]. γ-PGA elevated water-stable aggregate contents in potted soil used to grow spinach (*Spinacia oleracea* L.) [11].

Super absorbent polymers (SAP) have been developed as the materials with 3D network structure which allow for water absorption at an amount that is several hundred times higher than their original weight without dissolving [12, 13]. SAPs could be used in agricultural field. Studies have shown that the WOTE SAP (deionized water absorbance rate: 200 g/g) additions to the soil can reduce soil water leaching and improve the yields of maize [14]. The SAP (deionized water absorbance rate: 500–600 g/g) can potentially store a large amount of soil moisture, which could greatly increase the water use efficiency and the yield of potato tuber [15]. Mixing SAPs with soils could increase the soil porosity, make nutrients release slowly and reduce the soil bulk density. The Kehan 98 SAP (deionized water absorbance rate: 150–250 g/g) is able to elevate water-stable aggregate (at fraction size) proportion within soil [15]. It can be applied on plant seed surface to be the optimal coating materials and play an active role in transplantation of seedlings, cultivation without soil, cultivation of barren hills [16], and desert control. The SAPs could be roughly classified as synthetic polymers and natural-based polymers [17]. Synthetic SAPs are synthesized by acrylic acid, acrylamide, and other molecular monomers, which have the characteristics of high molecular weight and high water absorption rate [18]. However, SAPs synthesized by molecular monomers are not degradable and toxic [19]. Natural-based SAPs could be grafted or crosslinked with various natural polymers such as chitosan [20], cellulose [21], starch [22] and others. Natural-based SAPs have attracted much attention due to their ecofriendly nature. The synthesis of biodegradable and non-petroleum SAPs will be the focus of attention. The degradability of SAP based on γ-PGA is better than that of commonly used poly-acrylic acid SAPs which are cross-linked by C-C bond polymerization [23]. γ-PGA SAP is also a 3D network structure absorbent polymer formed by crosslinking the one-dimensional structure of γ-PGA with a crosslinking agent or itself [24, 25]. Additionally, their degradation products are γ-PGA and glutamic acid which can be utilized as fertilizers. The application of γ-PGA SAP will be more widely due to its good biodegradability and environmental friendliness with the increasing attention to the environment.

We have noticed that there are some studies on the effects of γ-PGA on crop yields in agricultural experiments, but there are few studies on the effect of γ-PGA on soil hydro-physical properties. Moreover, γ-PGA SAP is a novel degradable SAP, and there is little knowledge on its effects on soil hydro-physical properties. Therefore, the present study aimed to explore the effect of γ-PGA and γ-PGA SAP on the sandy loam soil hydro-physical properties such as infiltration, soil water retention characteristics, soil evaporation, together with soil porosity. And this will provide guidance for the recommended use of γ-PGA and γ-PGA SAP in agricultural application.

## Materials and methods

### Materials for experiment

#### Soil samples

In November 2017, soil was sampled from one field in Zhoujiabao Village, Xi'an City of Shaanxi Province (34°34′N; 108°52′ E) in the 0–30 cm tillage layer. The soil sample was subjected to air drying and filtering using the 2-mm mesh sieve. Soil EC and pH values were 562 us cm^-1^ and 7.95, respectively. The percentages of sand, silt, and clay were 49.85%, 43.05%, and 7.10%, respectively, for the sandy loam soil in accordance with the soil taxonomy released by the United States Department of Agriculture [26].

#### Sources of γ-PGA as well as γ-PGA SAP

**Fig 1:**
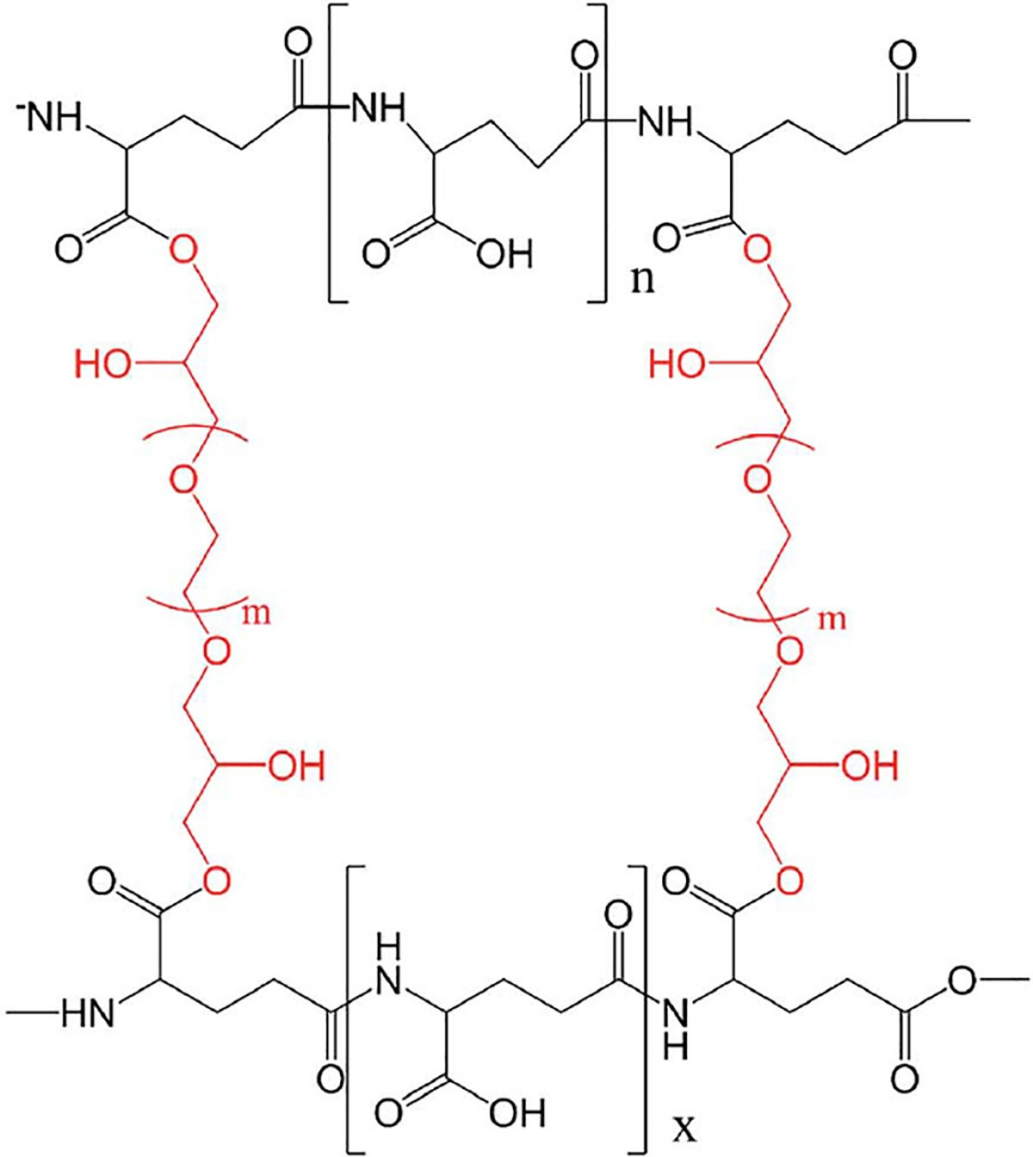
Molecular structure of γ-PGA and γ-PGA SAP. Note: The black part of figure represents γ-PGA, the red part is the crosslinking agent poly (ethylene glycol) diglycidyl ether (PEGDE), and the entire diagram illustrates the structure of γ-PGA SAP.

The molecular weight (MW) of the agricultural γ-PGA was approximately 1 million, and was purchased from Shineking Biotechnology (Nanjing, China). Fig 1 shows γ-PGA molecular structure (black color) [27].

The γ-PGA used as the primary raw material for making γ-PGA SAP was acquired from Shineking Biotechnology (Nanjing, China), and its MW was around 1 million. The preparation process of γ-PGA SAP was as follows. First of all, the distilled water (pH, 4.9) was used to dissolve γ-PGA at the 160 g L^-1^ final concentration. Then, poly (ethylene glycol) diglycidyl ether (PEGDE), which used as a crosslinking agent that contained 20% γ-PGA (mass weight), was blended with the above solution, followed by uniform stirring. The viscoelastic yellow colloid of cross-linked polymer was washed with distilled water and absolute ethanol for several times in succession for removing PEGDE and the non-reacted γ-PGA after crosslinking at 45°C for 48 h. The washed viscoelastic colloid was cut up, followed by overnight dehydration with absolute ethanol. After crushing the dehydrated pieces using a pulverizer, the obtained sample was filtered using the 2 mm mesh sieve, and transferred into the reagent bottle until it turned a white color, followed by final drying within the 60°C oven [28]. Fig 1 shows the γ-PGA SAP molecular structure (the entire diagram). Each gram of γ-PGA SAP could absorb 45.47 g g^-1^ and 651 g g^-1^ of water in 0.9% NaCl solution and distilled water, respectively.

**Fig 2:**
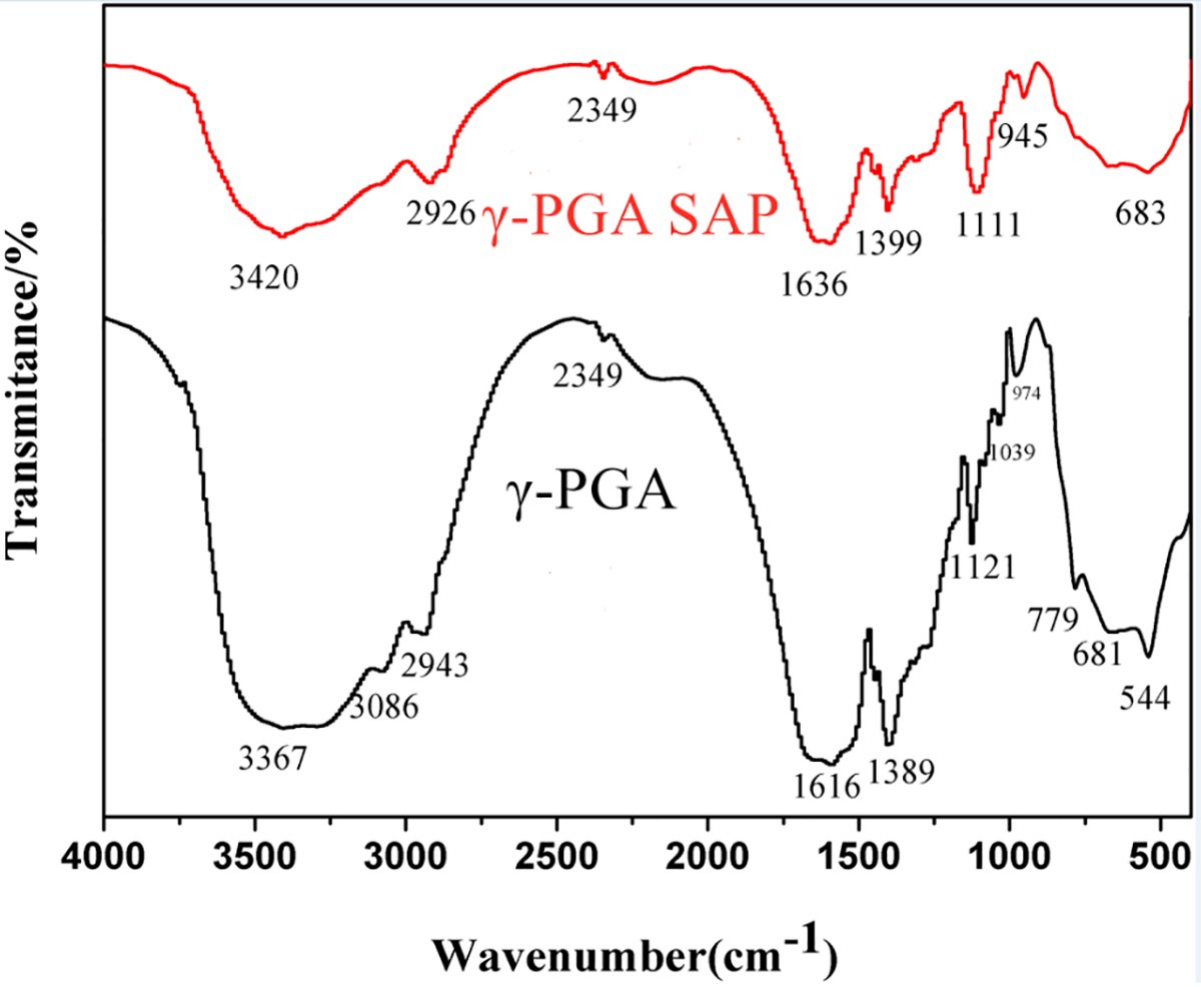
FTIR spectra of γ-PGA and γ-PGA SAP.

As shown in Fig 2 of the γ-PGA FTIR spectroscopy, the broad peaks observed at 3367 cm^-1^ and 3086 cm^-1^ arise from O–H and N–H stretching vibrations, respectively; that located at 2943 cm^-1^ was associated with C-H stretching vibration; that at 1616 cm^-1^ belonged to C = O stretching in carbony; that at 1039 cm^-1^ was caused by the stretching vibration of C-O. Compared with γ-PGA FTIR spectroscopy, γ-PGA SAP FTIR spectroscopy shows that the stretching vibration of N-H and the band strength of C = O stretching in carbonyl group are weakened, and the peak shape is narrowed and moves towards to high frequency. The peak of 1111 cm^-1^ stands for the stretching vibration of C-O-C, which is the characteristic group of γ-PGA SAP, and it is the strongest vibration peak in this region. Therefore, it illustrated interaction of PEGDE with γ-PGA, which results in new ester bond formation to be the link of crosslinking.

### Experimental methods

The addition different mass ratios regarding γ-PGA as well as γ-PGA SAP to sandy loam soil were 0, 0.05%, 0.10%, 0.15% and 0.20%, respectively. The effect of the different treatments on soil water infiltration, soil water retention characteristics, soil evaporation, soil expansion and soil porosity were studied by the following laboratory experiments.

#### Infiltration experiment

Soil samples of the different treatments were added to the columns (height, 60 cm; diameter, 8 cm), respectively, followed by division to ten layers (5 cm/layer) at the 1.44 g cm^-3^ density. The soil height was 50 cm and the layers were scraped to ensure that the column was filled evenly with soil during the filling process. The column bottom was the poriferous plexiglass plate covered with filter paper for preventing the loss of soil particles during infiltration. Water on soil column surface was maintained at 2 cm in depth and the Mariotte bottle was transparent plexiglass (inner diameter, 5 cm). In the case of 40 cm infiltration distance, the infiltration experiment ended.

#### Soil water retention characteristic measurement

The soil water retention characteristics (SWRCs) were determined by the centrifuge method (H-1400pf, Japan). The soil samples treated by different γ-PGA and γ-PGA SAPs rates were compressed to the 100 cm^3^ cutting rings, with a bulk density designed being 1.44 g cm^-3^, followed by 48 h of saturation within distilled water. Later, those saturated soil samples were transferred to the centrifuge for determining pressure head and soil water content (SWC). The speed, pF, along with equilibration time for each test is shown in Table 1.

**Table 1:** The speed and equilibrium time for each tested pressure head (h) on centrifugation (H-1400pf, Japan).

Rotation speed (rmp)	Pressure head (cm)	pF	Equilibrium time (min)	Rotation speed (rmp)	Pressure head (cm)	pF	Equilibrium time (min)
200	8.3	0.92	8	3100	1995.5	3.3	50
400	33.9	1.53	12	4400	4073.8	3.61	65
700	102.3	1.88	15	5300	5888.4	3.77	70
1000	208.9	2.32	25	6200	8128.3	3.91	75
1400	407.4	2.61	30	6900	10000	4	85
1700	602.6	2.78	38	7900	13182.6	4.12	90
2200	1023.3	3.01	45	8500	15135.6	4.18	90

#### Soil volume expansion rate and evaporation experiment

Soil samples of about 800 g of the different treatments were filled into soil columns (soil height filled, 14.5±0.2 cm; inner diameter, 7.04 cm), and every treatment was repeated thrice. The amount of water added to each soil column was the field water capacity determined by the SWRCs due to the field water capacity of each treatment was different. The internal heights of the soil in the columns were measured with Vernier calipers 24 h after adding water, and the soil columns were weighed and then placed into an incubator at 50°C. Soil column mass was recorded at 8:00 am and 8:00 pm every day. Evaporation experiment lasted for 14.5 d. The internal heights of the soil layers were recorded again at the end of evaporation experiment.

#### Soil porosity experiment

The γ-PGA, together with γ-PGA SAP, was blended with soil at 0.20%, respectively, and the control group (CK) was added without additives. The soils of different treatments were filled into the columns (height, 15 cm; diameter, 7.06 cm) and classified as two layers (6 cm/layer) at the 1.44 g cm^-3^ density. The height of soil layer was 12 cm and the layers were scraped to ensure that the column was filled evenly with soil in filling. Water depth on the surface of soil column was maintained at 2 cm, making the soil sample fully saturated. And then the soil columns were placed in the ventilation place and evaporate naturally. The CT scan image was performed using the GE phoenix v|tome|x m (General Electric Company) at the energy levels of 210 kV and 210 uA when the soil sample was dried. The voxel size for each image was 47.5um ×47.5um ×47.5um.

Those reconstructed images were rescaled using VG Studio MAX 2.2 (Germany), which were exported at a radiometric resolution of 16 bits. All images (676×676 pixels) were subsequently cut by ImageJ software (version, 1.39) for excluding area beyond soil column in order to avoid the influence of the boundary. The soil column was divided into 5 layers for statistical analysis in order to analysis the porosity of different soil layers. There are about 420 pictures of each soil layer, and the images of soil layer which is greatly influenced by the boundary between the surface layer and the bottom layer is removed.

The visualization, processing and analysis of images were carried out through commercial software AVIZO 9.0 [29]. The data was denoised with the median filter, which was the frequently adopted approach to process images for noise reducing and edge retaining [30, 31]. Edge enhancement was performed by adjusting the image contrast. After carefully comparing the initial images with the processed ones, individual threshold was screened for every image stack by the use of certain slices at diverse volume depths, for the sake of isolating pore phase away from soil [32]. According to segmentation of images, soil porosity was calculated and observed by the Avizo modules of *Volume Fraction* and *Volume Rendering*.

### Calculation methods

#### Infiltration model

The impacts of γ-PGA and γ-PGA SAP on soil water infiltration were analyzed and depicted using the Kostiakov infiltration model, as well as Philip infiltration model, respectively [33].

*(1) The Kostiakov infiltration model*. It represents the basic empirical infiltration model [34]. None of the parameters in this model has definite physical significance, this model has been extensively adopted due to its simplicity and easy calculation.

$$I=at^{1-b}$$ (1)

where *I* stands for the accumulated volume of infiltration, cm; *t* indicates time of infiltration, min; while *a*, *b* stand for the empirical coefficients.

*(2) The Philip infiltration model*. This model [35] has been extensively adopted for 1D longitudinal infiltration process with a uniform initial water content distribution, because it is simple and has definite physical significance.

$$I=St^{0.5}$$ (2)

where *I* stands for accumulated volume of infiltration, cm; *t* indicates time of infiltration, min; and *S* indicates the rate of infiltration, cm min^-0.5^.

#### Van Genuchten model

Soil water retention curves were fitted and calculated based on the VG model [36], the equation as follows:

$$\theta =\theta_{r}+\frac{\theta_{s}-\theta_{r}}{{\left[1+{\left(\alpha h\right)}^{n}\right]}^{m}}\;(m=1-\frac{1}{n},0<m<1)$$ (3)

In the above equation, *θ* stands for volumetric SWC, cm^3^ cm^-3^; *θ*_*r*_ represents residual SWC, cm^3^ cm^-3^, the residual SWC of this experiment is 0.078 cm^3^ cm^-3^; *θ*_*s*_ stands for saturated SWC, cm^3^ cm^-3^; *α* represents the intake value-related coefficient; n and m are the shape coefficients, whereas $m=1-\frac{1}{n}$. Field water capacity (FC) and permanent wilting point (PWP) are the SWC at *h* of 33 kpa and 1500 kpa, respectively. The total available water (TAW) is the SWC between FC and PWP, cm^3^ cm^-3^.

#### Soil water absorption of γ-PGA SAP

The soil water absorption of γ-PGA SAP is calculated by the following formula:

$$w_{SAP}=\frac{\theta_{sSAP}-\theta_{sSoil}}{m_{SAP}\gamma_{Soil}}$$ (4)

where, *w*_*SAP*_ is the soil water absorption of γ-PGA SAP in the soil, g g^-1^; *θ*_*sSAP*_ is the saturated soil water content of the treatments adding γ-PGA SAP in the soil, cm^3^ cm^-3^; *θ*_*sSoil*_ is the saturated soil water content of the CK, cm^3^ cm^-3^; *m*_*SAP*_ is the mass fraction of γ-PGA SAP in the soil, %; *γ*_*Soil*_ is the bulk density of the treatments, g cm^-3^. In this experiment, it is considered that the soil water absorption was the ratio of the soil water increased of the treatment with the addition of γ-PGA SAP to CK.

#### Evaporation rate

Evaporation rate was calculated as:

$$E=M\times 10/(\pi r^{2}\times 12)$$ (5)

In the above equation, *E* represents hourly evaporation in every soil column, mm h^-1^; *M* indicates mass alteration in every soil column at a 12-h measuring interval, g; *r* indicates soil column radius, cm.

#### Soil porosity

The soil porosity was determined according to the following equation [29]:

$$P(\%)=\frac{V_{p}}{V_{t}}\times 100$$ (6)

where *V*_*p*_ is soil pore volume, whereas *V*_*t*_ stands for volume of soil samples. *V*_*p*_ and *V*_*t*_ can be derived through *Volume Fraction* of Avizo 9.0.

## Results

### Effects of γ-PGA and γ-PGA SAP on the accumulated infiltration as well as infiltration rate

**Fig 3:**
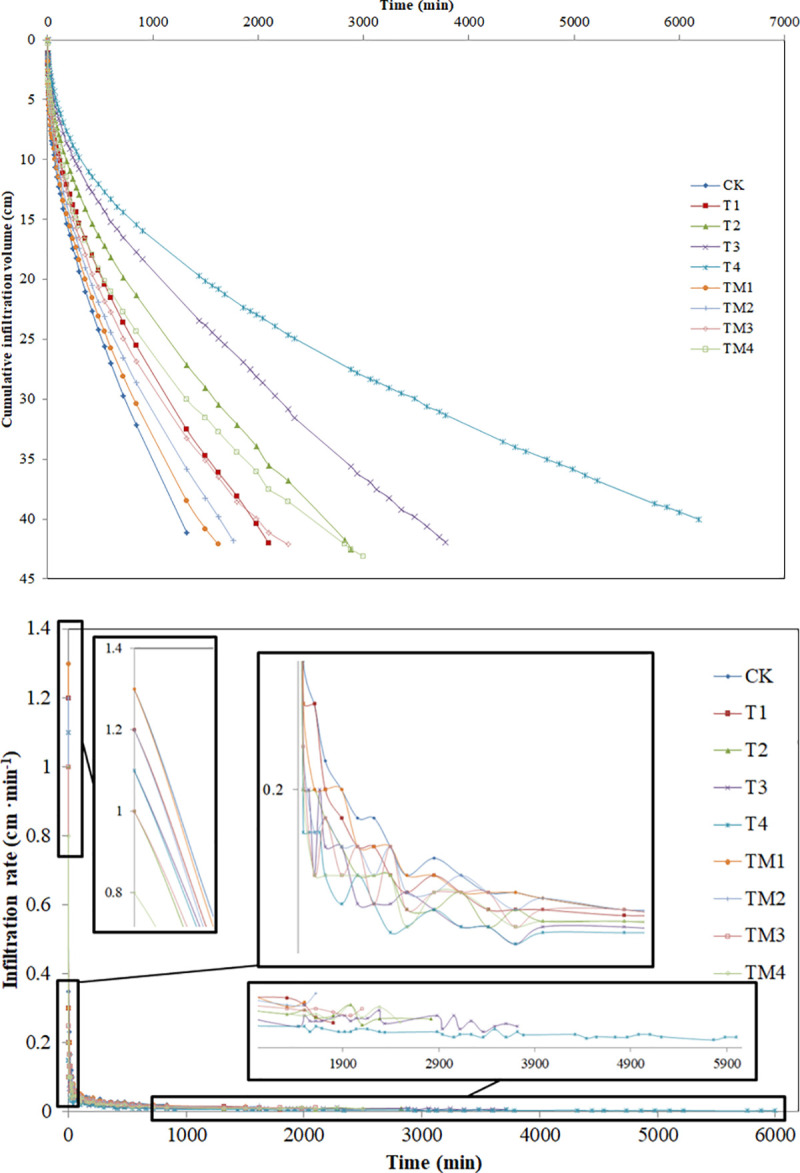
Effect of different contents γ-PGA and γ-PGA SAP added to a sandy loam soil on cumulative infiltration (a) and infiltration rate (b). Note: CK, check treatment; T1, T2, T3 and T4, treatments with 0.05%, 0.10%, 0.15% and 0.2% γ-PGA added, respectively; TM1, TM2, TM3 and TM4 treatments with 0.05%, 0.10%, 0.15% and 0.2% γ-PGA SAP added, respectively.

Fig 3(A) shows the water accumulated infiltration on sandy loam soil with the different addition amount of γ-PGA and γ-PGA SAP. The accumulated infiltration under each treatment increased with infiltration time prolonged. Cumulative infiltration after 1320 min decreased by 20.92% (T1), 34.06% (T2), 44.74% (T3), 54.65% (T4), 6.33% (TM1), 12.90% (TM2), 19.22% (TM3) and 27.01% (TM4) compared with CK, respectively, which suggested that the accumulated soil water infiltration at a given time after infiltration begins can be noticeably reduced by adding γ-PGA and γ-PGA SAP into soil. When equal amounts of γ-PGA and γ-PGA SAP were added, the accumulated soil water infiltration at a given time after infiltration began was much less for the γ-PGA treatments than for the γ-PGA SAP treatments.

The infiltration rate is the amount of soil water infiltrated into the soil per unit surface area of soil within a unit time, and is affected by factors such as soil texture, pore condition, and water supply intensity [37]. The effects of different γ-PGA and γ-PGA SAP addition amount on infiltration rate are shown in Fig 3(B). Infiltration rate fluctuations were likely due to errors in reading the Mariotte bottle scale and time step [38]. The infiltration rate showed rapid decline at an early experimental stage and the infiltration rate gradually stabilized as the experiment progressing. The stable infiltration rates of different treatments at the end of infiltration experiment decreased by 54.67% (T1), 56.44% (T2), 68.00% (T3), 84.00% (T4), 32.00% (TM1), 35.99% (TM2), 58.67% (TM3) and 61.60% (TM4) compared with CK, respectively. It is obvious that adding γ-PGA and γ-PGA SAP reduced the infiltration rate of soil water. Moreover, the infiltration rate for adding γ-PGA was noticeably lower than that for the addition of equivalent γ-PGA SAP to the soil.

#### Effects of γ-PGA and γ-PGA SAP on the parameters of infiltration

Both Philip and Kostiakov infiltration models were adopted for fitting those infiltration data observed (Table 2). The coefficients of determination (*R*^*2*^) values were >0.99. Soil infiltration rate for the Philip model depends on the liquid absorbing or releasing capacity of soil water via the capillary force. As γ-PGA and γ-PGA SAP addition amounts increased, the parameter ‘*S*’ in Philip model decreased, indicating that the capillary force weakened in its ability to absorb soil water. The coefficients of determination for the Kostiakov empirical models increased compared with those of Philip model. γ-PGA and γ-PGA SAP addition amounts affect the values of model parameters ‘*a*’ and ‘*b*’ in the Kostiakov model with different extent.

**Table 2:** Fitting parameters for Philip and Kostiakov infiltration models.

Treatment	Philip infiltration model		Kostiakov infiltration model		
	Infiltration rate (*S*)	*R*^*2*^	Empirical coefficient (*a*)	Empirical coefficient (*b*)	*R*^*2*^
CK	1.130	0.997	1.476	0.544	0.996
T1	0.899	0.998	0.974	0.512	0.998
T2	0.764	0.998	0.707	0.489	0.998
T3	0.652	0.996	0.498	0.465	0.998
T4	0.513	0.999	0.642	0.528	0.999
TM1	1.063	0.999	1.333	0.535	0.998
TM2	1.001	0.999	1.244	0.533	0.998
TM3	0.915	0.999	1.180	0.536	0.999
TM4	0.819	0.998	1.132	0.545	0.999

Note: CK, check treatment; T1, T2, T3 and T4, treatments with 0.05%, 0.10%, 0.15% and 0.2% γ-PGA added, respectively; TM1, TM2, TM3 and TM4 treatments with 0.05%, 0.10%, 0.15% and 0.2% γ-PGA SAP added, respectively.

### Effects of γ-PGA and γ-PGA SAP on SWRCs

The parameters of VG model for the different treatments by fitting with RETC software were given in S1 Fig and Table 3. As discovered, SWRCs with the addition of γ-PGA as well as γ-PGA SAP can be well fitted by the VG model, and the fitting degree is above 0.98. The TAW of T2 was the highest in the γ-PGA addition and CK treatment, but γ-PGA incorporated into the soil don’t significantly affected FC and total available water (TAW) compared with CK. In contrast, after γ-PGA SAP was added into soil, the FC remarkably increased to 29.6% (TM1), 34.0% (TM2), 36.9% (TM3) and 42.7% (TM4), respectively, compared with CK. The FC values were increased by 10.44%, 26.87%, 37.69% and 59.33%, respectively, over CK. And the TAW of addition of γ-PGA SAP was increased by 21.37% (TM1), 52.67% (TM2), 69.47% (TM3) and 111.45% (TM4), over CK. Therefore, with the increase in γ-PGA SAP addition, more water was stored in the γ-PGA SAP, resulting in increased FC and TAW. TM3 had the highest soil water absorption among the γ-PGA SAP addition treatments, and this was possibly associated with the change of soil volume.

**Table 3:** Effects of γ-PGA and γ-PGA SAP on parameters of van Genuchten model and total available soil water content.

Treatment	Paremeters of VG model				Volumetric soil water content (cm^3^ cm^-3^)			*w*_*SAP*_ (g g^-1^)
	*θ*_*s*_	α	n	R^2^	Field water capacity	Permanent wilting point	Total available water	
CK	0.447	2.567×10^−2^	1.303	0.984	0.268e	0.137c	0.131e	-
T1	0.461	3.100×10^−2^	1.290	0.988	0.271e	0.141c	0.130e	-
T2	0.475	2.868×10^−2^	1.301	0.986	0.277e	0.140c	0.137e	-
T3	0.453	2.996×10^−2^	1.292	0.985	0.268e	0.139c	0.128e	-
T4	0.452	3.01×10^−2^	1.295	0.987	0.265e	0.138c	0.127e	-
TM1	0.482	1.721×10^−2^	1.341	0.989	0.296d	0.137c	0.159d	49.01
TM2	0.521	1.1×10^−2^	1.381	0.988	0.340c	0.140c	0.200c	54.99
TM3	0.568	1.041×10^−2^	1.382	0.986	0.369b	0.147b	0.222b	55.97
TM4	0.602	0.634×10^−2^	1.425	0.986	0.427a	0.150a	0.277a	54.0

Note: Values followed by a different letter are significantly different at P < 0.05 according to a least significant difference (LSD) test in each column of volumetric soil water content. FC and PWP are the soil water content when h is equal to 33kpa and 1500kpa, respectively. CK, check treatment; T1, T2, T3 and T4, treatments with 0.05%, 0.10%, 0.15% and 0.2% γ-PGA added, respectively; TM1, TM2, TM3 and TM4 treatments with 0.05%, 0.10%, 0.15% and 0.2% γ-PGA SAP added, respectively.

### Effects of γ-PGA and γ-PGA SAP on evaporation rate

**Fig 4:**
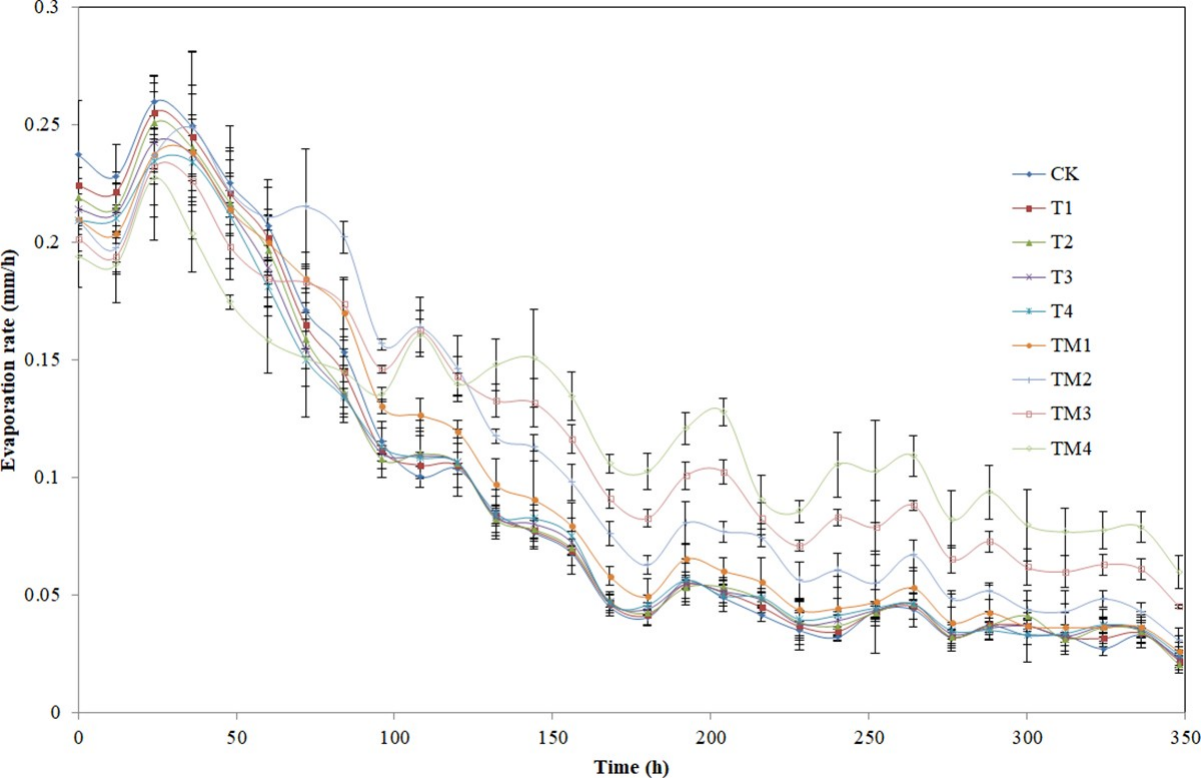
Effect of different contents of γ-PGA and γ-PGA SAP added to a sandy loam soil on the soil evaporation rate. Note: Error bars represent standard deviations.

Evaporation experiment was carried out when the soil water beginning at field water capacity. The evaporation rates of all different treatments generally decreased with time (Fig 4), which was caused by decreasing soil moisture and increasing suction force of soil water. Differences in evaporation rate were not significant between γ-PGA addition treatments and CK, and evaporation rate of γ-PGA addition treatment were lower than that of CK at initial evaporation stages. The evaporation rate of the treatments with γ-PGA SAP addition decreased compared with CK in the early evaporation stage, and that exceeded CK at 60 h (TM1), 72 h (TM2), 84 h (TM3), 96 h (TM4) under γ-PGA SAP addition treatments. The evaporation rate was higher with the increasing of the amount of γ-PGA SAP to the soil in the middle and later stages of the experiment.

### Effects of γ-PGA and γ-PGA SAP on SWC

**Fig 5:**
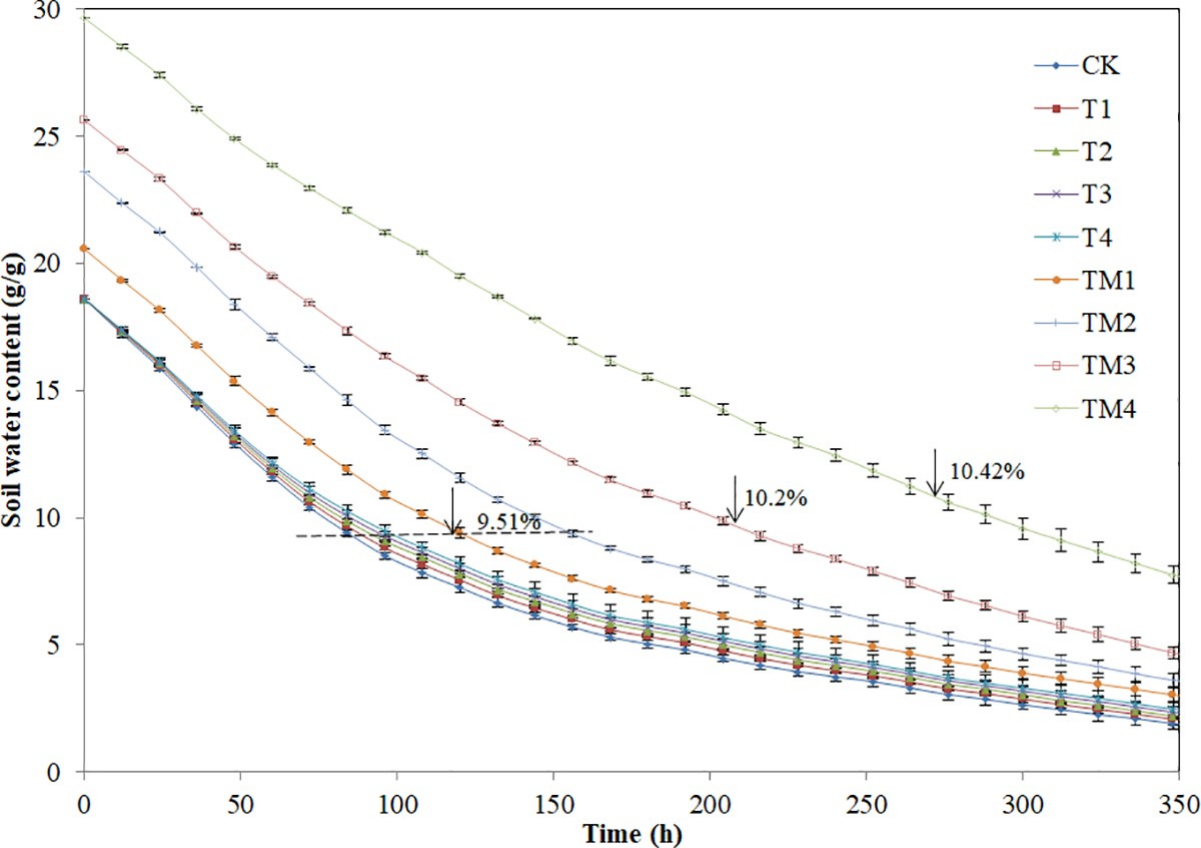
Effect of different contents of γ-PGA and γ-PGA SAP added to a sandy loam soil on the soil water content. Note: The soil water content is the soil mass water content, g g^-1^. Error bars represent standard deviations.

The T1 to T4 treatments with γ-PGA addition slightly prolonged the retention time for SWC from FC to PWP compared with CK (Fig 5), but the differences were not significant. The time for SWC of γ-PGA SAP addition treatment to decline from FC to PWP was 1.48 times (TM1), 1.88 times (TM2), 2.01 times (TM3) and 2.87 times (TM4) longer than that of CK.

### Effects of γ-PGA and γ-PGA SAP on soil expansion

At the beginning and the completion of evaporation experiment, soil column internal heights were measured, as shown in Table 4. As a result, the height of soil filling reduced by 0.22% (CK), 0.62% (T1), 0.41% (T2), 0.65% (T3) as well as 0.81% (T4) compared with that before water was added, but the changes were not significantly different among these three treatments. The height of the soil filling increased by 0.47%, 4.50%, 6.32% and 8.43% for the TM1, TM2, TM3 and TM4 treatments, respectively, after water was added. Upon the completion of experiment, soil filling height changed by -1.54% (CK), -1.54% (T1), -1.58% (T2), -1.98% (T3) and -2.15% (T4) respectively, compared with the height before adding water, and differences among diverse treatments were not significant. The height of the soil filling adding containing γ-PGA SAP increased by 0.17% (TM1), 2.45% (TM2), 4.72% (TM3) and 6.28% (TM4) compared with the height before adding water.

**Table 4:** Soil porosity of different soil layers in each treatment.

Soil depth	CK	T4	TM4
1–3 cm	2.25%b	3.30%b	24.73%a
3–5 cm	1.71%b	3.14%b	16.31%a
5–7 cm	0.74%b	0.93%b	9.92%a
7–9 cm	0.47%b	0.89%b	5.60%a
9–11 cm	0.16%b	0.37%b	4.21%a

Note: Values followed by a different letter are significantly different at P < 0.05 according to a least significant difference (LSD) test in each column. CK, check treatment; T4, treatments with 0.2% γ-PGA added, respectively; TM4 treatment with 0.2% γ-PGA SAP added, respectively.

### Effects of γ-PGA and γ-PGA SAP on soil porosity

The distribution of soil porosity in different soil layers of soil columns was showed in S2 Fig and Table 4. The soil porosity has the highest value in the topsoil of each treatment and it gradually decreases as the depth of soil layer increase. Compared with CK, the soil porosity of TM4 increased by 10.99 times (1–3 cm), 9.54 times (3–5 cm), 13.41 times (5–7 cm), 11.91 times (7–9 cm) and 26.31 times (9–11 cm). The soil porosity of each layer of TM4 was significantly higher than that of CK and T4. The soil porosity of T4 increased by 1.47 times (1–3 cm), 1.84 times (3–5 cm), 1.26 times (5–7 cm), 1.89 times (7–9 cm) and 2.31 times (9–11 cm), compared with CK. The soil porosity of T4 was slightly higher than that of CK, but there was no significant difference between the T4 and CK.

## Discussion

There are numerous carboxyls in the γ-PGA and γ-PGA SAP molecular structures, conferring upon them strong hydrophilic properties. γ-PGA is a water-soluble substance because of its single-chain molecular structure and hydrophilic groups. It can increase the viscosity of water and subsequently alter a number of soil properties after dissolving in water [39]. γ-PGA SAP is a water-insoluble three dimensional network structure modification of γ-PGA (Fig 1). Similarly, its molecular structure has many hydrophilic groups, and these hydrophilic groups can combine with water molecules to form hydrogen bonds. After forming a hydrogen bond with the water molecules, those polar groups within γ-PGA SAP will be ionized, which results in the mutual repelling of those negatively charged groups, then causes the expansion of the three-dimensional structure of γ-PGA SAP, and forms the osmotic pressure difference between γ-PGA SAP interior and exterior. Due to the osmotic pressure, the water molecules permeate and diffuse to γ-PGA SAP interior, thereby generating the phenomenon of water absorption and swelling for γ-PGA SAP, forming a hydrogel with viscoelastic properties [40], and thus changing the properties of soil.

The γ-PGA and γ-PGA SAP addition could reduce infiltration rate and cumulative infiltration of sandy loam soil, but the mechanisms of reducing soil infiltration rate and cumulative infiltration are different for the two polymers. γ-PGA could reduce the cumulative infiltration and infiltration rate by increasing the soil water viscosity and reducing the soil water transport rate [41], while the γ-PGA SAP could reduce the cumulative infiltration and infiltration rate by hydrogel swelling, filling soil pores and reducing the soil water migration path [42, 43]. Adding γ-PGA and γ-PGA SAP could slow down soil water infiltration in the infiltration process.

γ-PGA SAP addition remarkably increased sandy loam soil porosity (Table 4) because γ-PGA SAP swelling squeezes adjacent soil and γ-PGA SAP shrinks after the soil dries. However, the soil porosity after γ-PGA addition slightly increased, but there was no significant difference compared with CK (Table 4). It is the fact that the γ-PGA would be adhered to soil particles after dissolving in water and causes the increasing of soil porosity.

Additions of γ-PGA and γ-PGA SAP affected sandy loam soil expansion in a different way (Table 5). The soil volume of the control treatment exhibited a certain degree of shrink because soil porosity was decreased by the soil water’s action [43]. The volume of soil also decreased after adding γ-PGA, with no significant difference compared with the control group (Table 5). In contrast, γ-PGA SAP expanded when it absorbed water and squeezed the surrounding soil in the soil column, thereby significantly increasing the height of the soil compared with the control group.

**Table 5:** Sandy loam soil expansion under different applications of γ-PGA and γ-PGA SAP.

Treatment	Soil expansion before evaporation/%	Soil expansion after evaporation/%
CK	-0.22±0.26d	-1.54±0.41e
T1	-0.62±0.21d	-1.54±0.38e
T2	-0.41±0.33d	-1.58±0.76e
T3	-0.65±0.41d	-1.98±0.50e
T4	-0.81±0.21d	-2.15±0.43e
TM1	0.47±0.39d	0.17±0.29d
TM2	4.50±1.62c	2.45±1.46c
TM3	6.32±1.38b	4.72±1.46b
TM4	8.43±1.39a	6.28±1.17a

Note: Values followed by a different letter are significantly different at P < 0.05 according to a least significant difference (LSD) test in each column. Error bars represent standard deviations. CK, check treatment; T1, T2, T3 and T4, treatments with 0.05%, 0.10%, 0.15% and 0.2% γ-PGA added, respectively; TM1, TM2, TM3 and TM4 treatments with 0.05%, 0.10%, 0.15% and 0.2% γ-PGA SAP added, respectively.

0.10% γ-PGA addition improved TAW, but there was no significant difference between CK and the γ-PGA addition treatment. However, γ-PGA SAP addition significantly improved SWC, and FC and TAW elevated as γ-PGA SAP increased. This is mainly related to the characteristic of the two substances. The higher γ-PGA SAP addition increased the pore volume of the sandy loam soil due to the greater expansion of γ-PGA SAP, thereby maintaining more soil water in the soil. γ-PGA is a water-soluble substance, which has little effect on the soil pores, and thus has a little effect on the soil water holding capacity. The soil water absorption ratio was highest at the γ-PGA SAP addition amount of 0.15%, which might be caused by the influence of pore volume of soil as γ-PGA SAP addition amount increased. Adding γ-PGA SAP significantly increased FC, and also could significantly increase the soil porosity, but excessive porosity in the soil might adversely affect crop growth. Therefore, γ-PGA SAP could be buried in the deep soil which could avoid excessive soil porosity.

Adding γ-PGA might slightly prolong the time of the soil water change from FC to PWP during the evaporation experiment. The possible reason is that the increasing viscosity of soil water under γ-PGA addition treatment leads to reduced evaporation. However, adding γ-PGA SAP significantly prolonged the time of the soil water change from FC to PWP during the evaporation experiment, which is due to the γ-PGA SAP addition could remarkably improve FC and γ-PGA SAP could keep water under a certain of pressure and temperature [44].

The experiment was carried out in the laboratory, but there might be greater variance in the agricultural practical field. Therefore, they will be applied to the agricultural field experiment to study their effect on soil water retention, soil microorganism and crop yield in the future.

## Conclusions

In this paper, water-soluble agricultural γ-PGA and its derivative γ-PGA SAP were added to a sandy loam soil for investigating how the different addition amounts affected sandy loam soil hydro-physical properties. The conclusions are as follows:

Adding γ-PGA and γ-PGA SAP reduced accumulated water infiltration of the sandy loam soil, thus decreasing deep percolation loss of soil water. The cumulative infiltration with the addition of γ-PGA treatment was less than that the treatment with γ-PGA addition when the addition amounts were the same. The TAW was highest at the treatment of γ-PGA addition when the γ-PGA addition amount to the soil was 0.10%. The γ-PGA SAP addition amount dramatically maintained more soil moisture in the sandy loam soil. The γ-PGA SAP addition significantly prolonged the time from FC to PWP during the evaporation experiment. Thereby this study could provide the useful information for the agricultural application of γ-PGA as well as γ-PGA SAP for the agricultural production to sandy loam soil.

## Supporting information

S1 Fig Effect of different contents of γ-PGA and γ-PGA SAP added to soil on the soil water holding capacity. (DOCX)

S2 Fig The soil porosity of different soil depth. Note: The figure on the left of the soil porosity stands for CK; the figure on the middle of the soil porosity stands for T4; the figure on the right of the soil porosity stands for TM4.
(DOCX)
