# Supplementary material for: Effects of poly-γ-glutamic acid and poly-γ-glutamic acid super absorbent polymer on the sandy loam soil hydro-physical properties
Source: PLoS One. 2021 Jan 12;16(1):e0245365. doi: 10.1371/journal.pone.0245365 (PMC7983855; doi:10.1371/journal.pone.0245365)
Supplement: S1 Fig — (DOCX) [file pone.0245365.s001.docx]

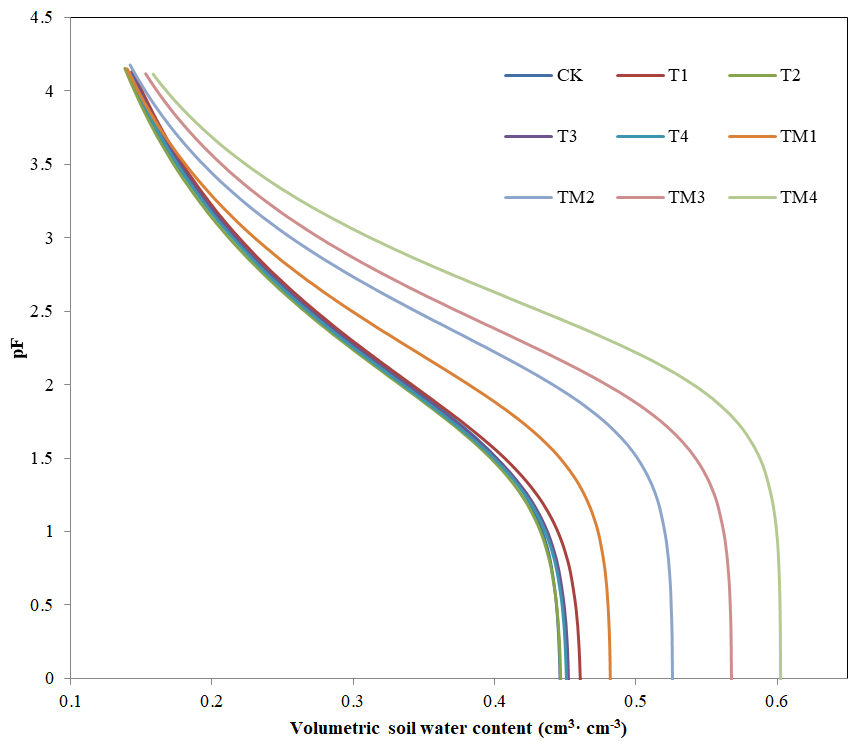


**S1 Fig.** Effect of different contents of γ-PGA and γ-PGA SAP added to soil on the soil water holding capacity
