# Supplementary material for: Effects of poly-γ-glutamic acid and poly-γ-glutamic acid super absorbent polymer on the sandy loam soil hydro-physical properties
Source: PLoS One. 2021 Jan 12;16(1):e0245365. doi: 10.1371/journal.pone.0245365 (PMC7983855; doi:10.1371/journal.pone.0245365)
Supplement: S2 Fig — Note: The figure on the left of the soil porosity stands for CK; the figure on the middle of the soil porosity stands for T4; the figure on the right of the soil porosity stands for TM4. (DOCX) [file pone.0245365.s002.docx]

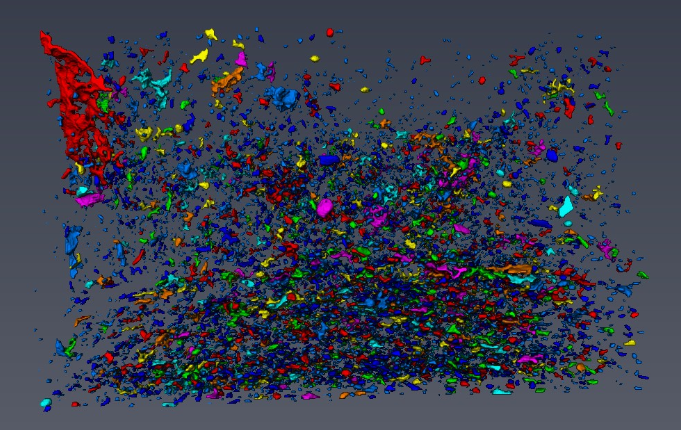

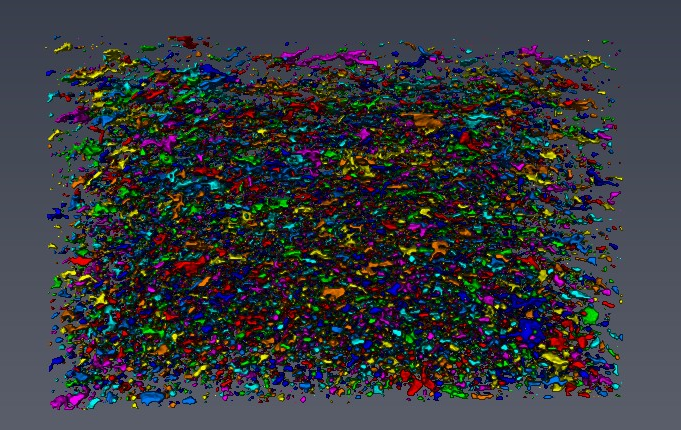

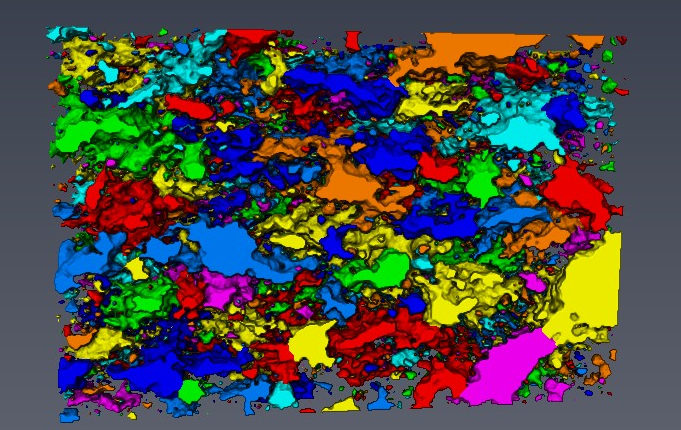


(a) 1-3 cm


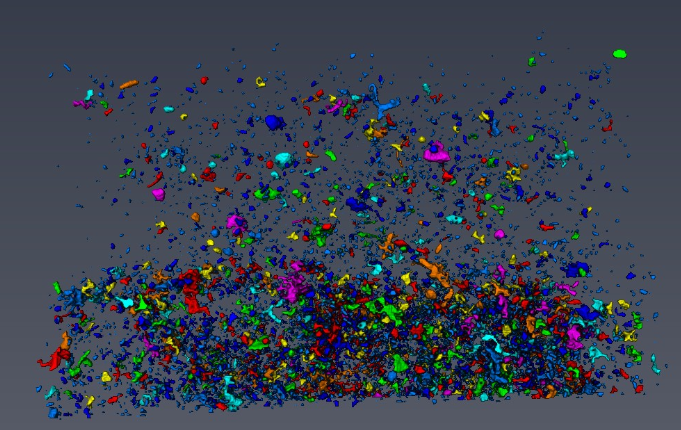

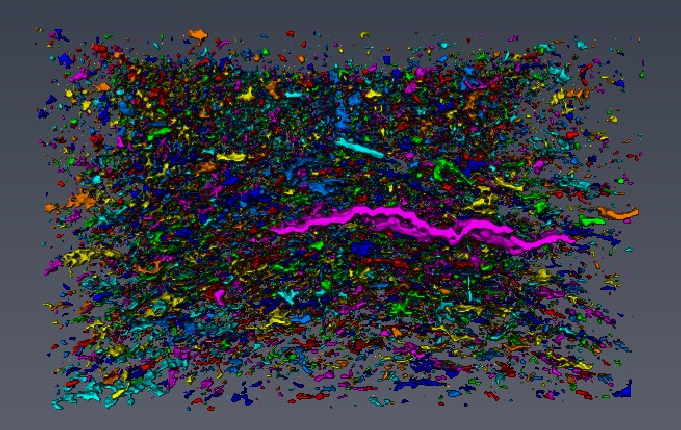

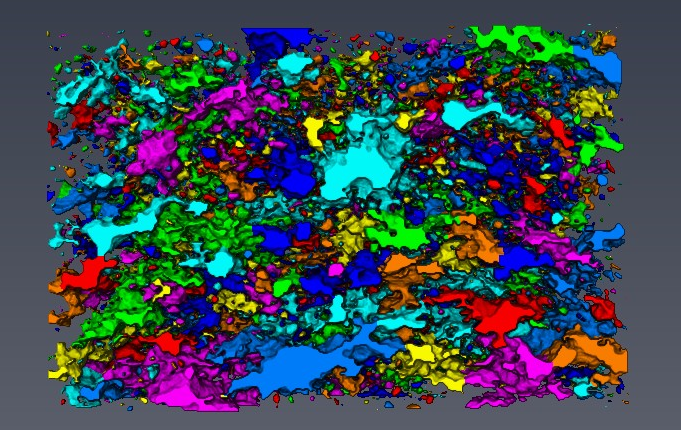


(b) 3-5 cm


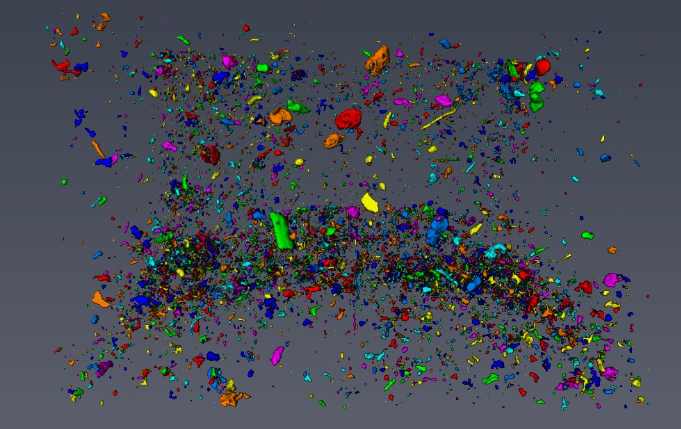

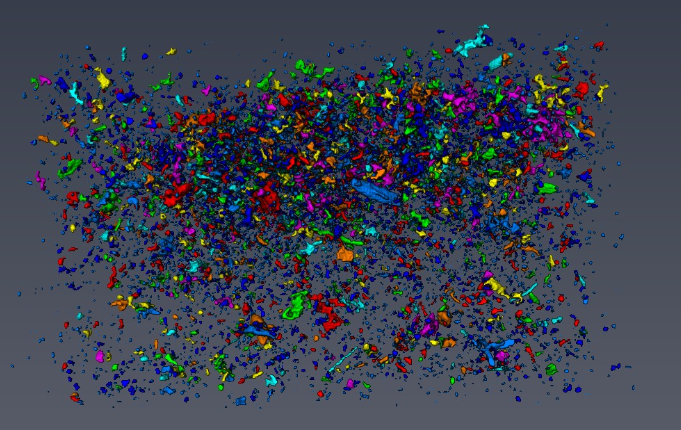

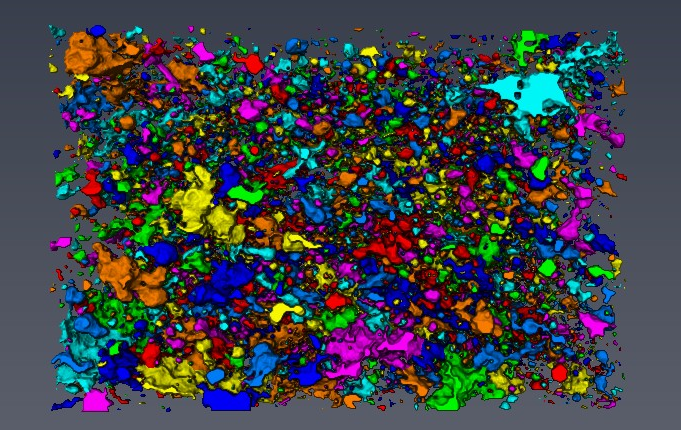


(c) 5-7 cm


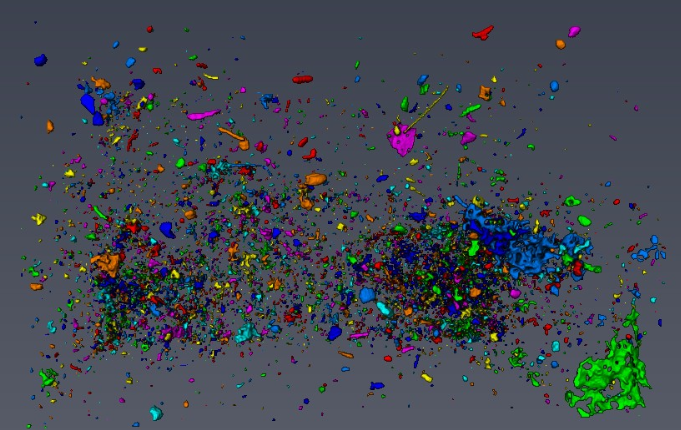

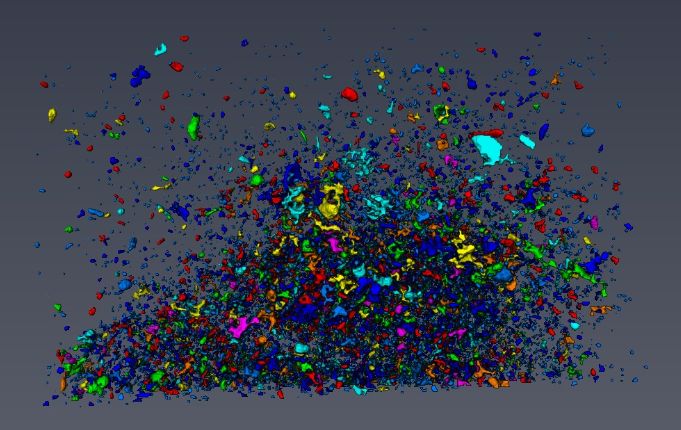

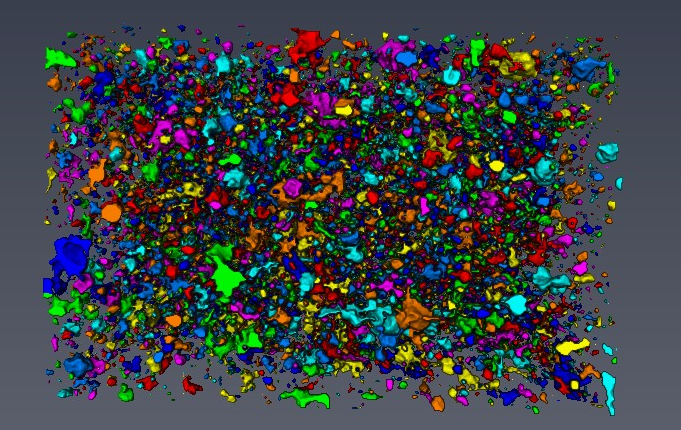


(d) 7-9 cm


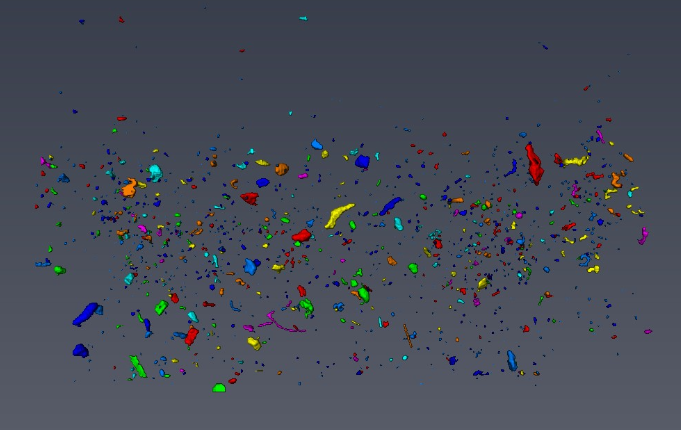

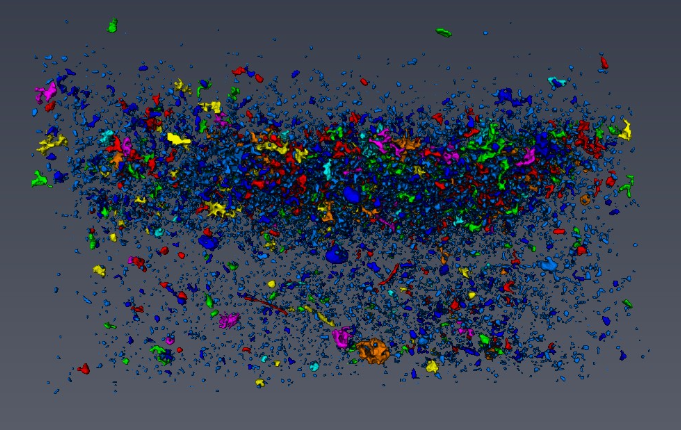

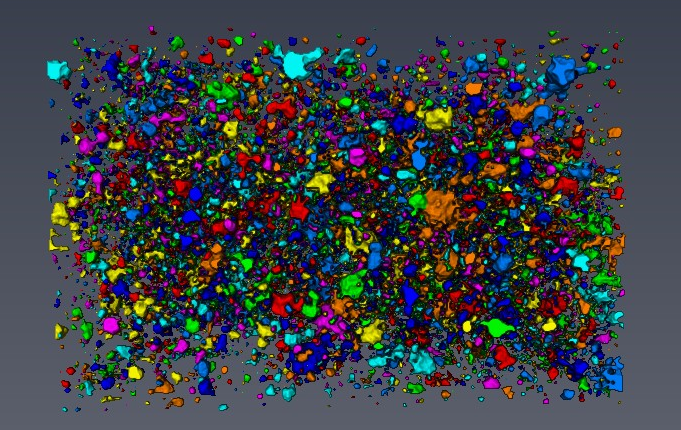


(e) 9-11 cm

**S2 Fig.** The soil porosity of different soil depth

Note: The figure on the left of the soil porosity stands for CK; the figure on the middle of the soil porosity stands for T4; the figure on the right of the soil porosity stands for TM4.
